# Health financing reforms for Universal Health Coverage in five emerging economies

**DOI:** 10.7189/jogh.11.16005

**Published:** 2021-11-20

**Authors:** Chris Atim, Indu Bhushan, Mark Blecher, Ramana Gandham, Vikram Rajan, Jonatan Davén, Olusoji Adeyi

**Affiliations:** 1 Results for Development, Accra, Ghana; 2 National Health Authority, New Delhi, India; 3 National Treasury of South Africa, Pretoria, South Africa; 4 Independent consultant, Nairobi, Kenya; 5 World Bank Group, Washington, D.C., USA; 6 Resilient Health Systems, Washington, D.C., USA

## Abstract

**Background:**

Many countries have committed to achieving Universal Health Coverage. This paper summarizes selected health financing themes from five middle-income country case studies with incomplete progress towards UHC.

**Methods:**

The paper focuses on key flagship UHC programs in these countries, which exist along other publicly financed health delivery systems, reviewed through the lens of key health financing functions such as revenue raising, pooling and purchasing as well as governance and institutional arrangements.

**Results:**

There is variable progress across countries. Indonesia’s Jaminan Kesehatan Nasional (JKN) reforms have made substantial progress in health services coverage and health financing indicators though challenges remain in its implementation. In contrast, Ghana has seen reduced funding levels for health and achieved less than 50% in the UHC service coverage index. In India, despite Ayushman Bharat (PM-JAY) reforms having provided important innovations in purchasing and public-private mix, out of pocket spending remains high and the public health financing level low. Kenya still has a challenge to use public financing to enhance coverage for the informal sector, while South Africa has made little progress in strategic purchasing.

**Conclusions:**

Despite variations across countries, therefore, important challenges include inadequate financing, sub-optimal pooling, and unmet expectations in strategic purchasing. While complex federal systems may complicate the path forward for most of these countries, evidence of strong political commitment in some of these countries bodes well for further progress.

Global declarations and country commitments, such as that of the United Nations General Assembly on Universal Health Coverage (UHC) [1], have put UHC at the centre of health policies and strategies [2]. While some countries have already achieved UHC, most are still striving for it, progress is uneven across countries, and there are gaps in knowledge about how to successfully move from policy to achievement amidst resource constraints [3,4]. Several papers including the landmark World Health Report of 2010 have focussed on the importance of health financing for UHC [5]. The World Health Organization (WHO) has developed a useful tool for assessing progress on health financing [6]. Some previous studies have examined bottlenecks to progress in achieving UHC and proposed strategies for unblocking these [7]. However, it is unclear to what extent these bottlenecks have been resolved. The World Bank thus commissioned a set of country cases to assess recent progress towards UHC. To this end, the article by Preker et al in this edition portrays several countries such as South Korea and Turkey, which have made substantial progress to achieving UHC [8]. In contrast, the five countries for which case studies were commissioned in this paper portray varying degrees of incomplete progress to UHC.

The aim of this paper is to contribute to closing the gaps in knowledge by synthesizing information along key themes from five case studies of middle-income countries – Ghana, India, Indonesia, Kenya, and South Africa – with political commitment to and some progress toward UHC, but where UHC systems are still in the process of development and face specific challenges. Each case highlighted here is different from the others in some key areas, but the selected themes help to highlight some common issues, challenges, and opportunities relevant to these countries.

## METHODS

Five country case studies were commissioned to illustrate recent and contemporary experiences in mission-critical themes that drive progress toward UHC. A writing group constituted of experts with experience of UHC and health financing reform from each country worked on the case studies. Given space limitations, this paper focuses on selected health financing dimensions of UHC. The authors used the WHO framework on health financing [9] to guide both data collection and analysis. The framework identifies the key health financing functions as revenue raising, pooling and purchasing, to which the writing team added the governance and institutional arrangements because of the importance of this dimension to some of the case studies – in terms of potential challenges and opportunities for progressing towards UHC.

Literature searches were done for each country, focusing on the specific common study themes cited below, combined with use of international repositories of country data. Data sources consulted for the analysis included internationally recognized databases such as the World Development Indicators, the Global Health Expenditure Database, and the World Health Statistics, as well as national data sources - National Health Accounts, legislation on the setting up and design of the UHC schemes of each country, national household health and other socio-economic surveys, sector and UHC scheme reviews and other secondary data sources.

This paper synthesises the five country case studies, using the following set of common themes to examine progress:

- Revenue raising and funding levels
- Financial and risk pooling
- Strategic purchasing
- Governance and institutional arrangements, including decentralisation.

## RESULTS

**Table 1** provides an introductory comparison of the five countries across key variables that are discussed in more detail in subsequent sections. Notable in several of these countries were low levels of public health financing often associated with sub-optimal levels of professional staffing and incomplete population coverage with low to modest scores on the UHC coverage index. Nevertheless, all of these countries are in the process of health sector reform and have good indicators in several important areas.

**Table 1:** Comparing five countries in transition [10-12]

Indicator	Year(s) measured	Ghana	India	Indonesia	Kenya	South Africa
**Economy:**						
Population million [10]	2018	29.8	1 352.6	267.7	51.4	57.8
GDP per capita US$ [11]	2019	2,221	2 098	4 197	2 004	5 978
GDP growth % [11]	2019	6.5	4.2	5.0	5.4	0.2
Fiscal deficit % [11]	2019	-7.3	-8.2	-2.2	-7.7	-6.3
Revenue/GDP ratio % [11]	2019	13.7	19.3	14.2	17.7	29.1
**Health expenditure:**						
GGHE-D per capita US$ [12]	2018	30.3	19.6	55.1	37.2	284.3
GGHE-D per capita PPP-adjusted US$ [12]	2018	65.3	74.2	185.1	75.5	610.4
GGHE-D PPP-adjusted average annual % growth [12]	2013-2018	-2.3	10.3	14.8	8.2	2.5
GGHE-D as % of GDP [12]	2018	1.4	1.0	1.4	2.2	4.5
CHE as % GDP [12]	2018	3.5	3.5	2.9	5.2	8.3
GGHE-D as % of GGE [12]	2018	6.4	3.4	8.5	8.5	13.3
Out-of-pocket as % CHE [12]	2018	37.7	62.7	34.9	23.6	7.7
**Human resources for health:**						
Doctors per 10 000 total population [10]	2010-2018	1.4	8.6	4.3	1.6	9.1
Nurse and midwifery personnel per 1000 population [10]	2010-2018	0.9	2.1	1.3	1.6	5.2
**Health coverage and health outcomes:**						
UHC service coverage index [10]	2017	47	55	57	55	69
Under 5 mortality rate (per 1000 live births) [10]	2018	48	37	25	41	34
Family planning coverage, women of reproductive age [10]	2010-2019	46.3	72.8	77.6	77.6	79.7
Maternal mortality ratio (per 100 000 live births) [10]	2017	308	145	177	342	119
Financial protection (% of pop with >10% household exp on health) [10]	2010-2018	1.1	17.3	2.7	5.4	1.4
Vaccine coverage % (measles 2nd dose) [10]	2018	83	80	67	45	50
Life expectancy at birth [10]	2016	63.4	68.8	69.3	66.7	63.6
Neonatal mortality rate (per 1000 live births) [10]	2018	24	23	13	20	11

CHE – current health expenditure, GGHE-D – domestic general government health expenditure, GGE – general government expenditure, GDP – gross domestic product, OOP – out-of-pocket payments, PPP – purchase power parity

**Figure 1:**
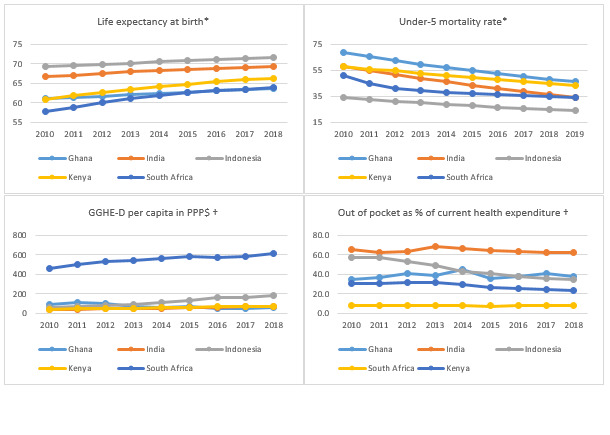
Key health outcomes and financing trends. **Panel A.** Life expectancy at birth. **Panel B.** Under-5 mortality rate. **Panel C.** Domestic general government health expenditure per capita in purchasing power parity adjusted US$. **Panel D.** Out-of-pocket expenditure as a percentage of current health expenditure: Sources: * [13], † [12].

All five countries showed some good results, consistent with their income status, on some health outcome indicators, such as under 5 mortality rates and life expectancy, whereas trends for health financing indicators, such as domestic general government health expenditure (GGHE-D) per capita and out-of-pocket payments (OOP) as % of total current health expenditure, differ between the countries (**Figure 1**). Indonesia has made significant progress with the low child mortality rates, significant growth in public health financing from a low base (**Figure 1**), and reduced reliance on OOP payments. In contrast, funding in Ghana has declined and progress with health outcomes (with the exception of financial protection) was comparatively slow. As Table 1 shows, coverage with services such as immunisation (except for Kenya and to some extent Indonesia) and family planning (except Ghana) was high. Indonesia and South Africa appear to have made particular progress with child mortality. The maternal mortality ratio (MMR) remained unacceptably high in Kenya and Ghana’s MMR was also well above its lower middle-income country (LMIC) group average (253).

Despite improvements in some health indicators presented in **Table 1**, service coverage index in most of the countries was still lower than ideal (47% Ghana, 57% Indonesia, 55% India and Kenya).

**Table 2** provides a brief overview of the UHC programmes in the five countries for each of the dimensions examined in this paper.

**Table 2:** Country program summary by theme

Theme	Ghana	India	Indonesia	Kenya	South Africa
**Revenue raising**	Public taxes – value-added tax (VAT)/sales tax, social security. Premiums -<4% of income	Publicly financed Ayushman Bharat UHC scheme (National and state Governments)	Payroll taxes for formal sector, voluntary premiums for non-poor informal sector and public subsidies for poorest	Mandatory formal sector premiums; voluntary informal sector; national and county governments	Currently general taxes in public sector and premiums for private medical schemes. Plans to move towards greater public share of health financing as part of National Health Insurance (NHI) reform.
**Financial and risk pooling**	UHC scheme (National Health Insurance Scheme – NHIS) covering approx. 40% of population; unified benefit package with no fragmentation in UHC scheme	National scheme but with significant role for states in implementation, including ability to vary design elements and add on benefits. Just over half (700 m out of 1300 m people) covered; States-based implementation involves level of fragmentation	Single UHC scheme, Jaminan Kesehatan Nasional (JKN), covering >215m people or 81% of pop. in 2019; uniform benefit package	National Hospital Insurance Fund (NHIF) for formal sector, national government subsidies for vulnerable (elderly and disabled); and informal sector making voluntary contributions. County UHC pilots; 20% pop. coverage	In principle, public sector covers entire population pooled in 9 provincial governments, but with access and quality challenges. Voluntary health insurances (VHIs) cover 17% of population through fragmented risk pools. NHI aims to cover entire population and VHIs transitioning towards complementary role
**Strategic purchasing**	Single purchaser; Clinical audits for quality checks; relies on inefficient diagnosis-related groups (DRGs) and fee-for-service (FFS) for provider payments; capitation abandoned for political reasons; broad benefit package not well-aligned with resources and health priorities; data use not well developed.	Ayushman Bharat provides comprehensive primary, secondary and tertiary care but states can add packages on top of guaranteed base package. Standard treatment guidelines for most of packages prepared to enforce accountability. States use different payment modalities: Some use insurance companies; others use trusts and still others use a mixed payment model which involves both of those modalities. The IT system is used to verify compliance with guidelines before making payments.	Single purchaser (*Badan Penyelenggara Jaminan Sosial – Kesehatan* or BPJS – K); strategic purchasing functions divided between BPJS – K and the Ministry of Health (MOH); no co-payments or global caps on expenditures; some evidence of adverse selection; fragmentation of information systems, weak accountability of local governments and providers for quality and open-ended hospital payment systems; data quality and use still poor.	Multiple benefit packages – over 80 – and high operational costs impacting NHIF efficiency. Non retention of fee income by public hospitals disincentivizes revenue collection; NHIF has high operational costs; good data use by NHIF but challenges at county levels.	Virtually no strategic purchasing in public and private sector. Public sector uses incremental budgets, and private sector mainly FFS. Plans to use capitation (primary health care) and DRGs (hospitals) under NHI.
**Governance and institutional arrangements, including decentralisation**	Management initially decentralized to district levels, but NHIS recentralized as one national scheme in 2012	Decentralized to 36 states for implementation: allowing flexible adaptations and no ‘one size fits all’ approach	Decentralization here has strained the capacity of local governments to do more integrated health planning and budgeting	NHIF has established offices at county level but counties not well represented on its Board.	Decentralised with provinces responsible for health provision. NHI aiming to centralise funding/purchasing under national NHI Fund.
				Delivery of essential health services decentralized to 47 County Governments.	

## DISCUSSION

### Financing of UHC: Inadequate funding levels for health and UHC

**Figure 2:**
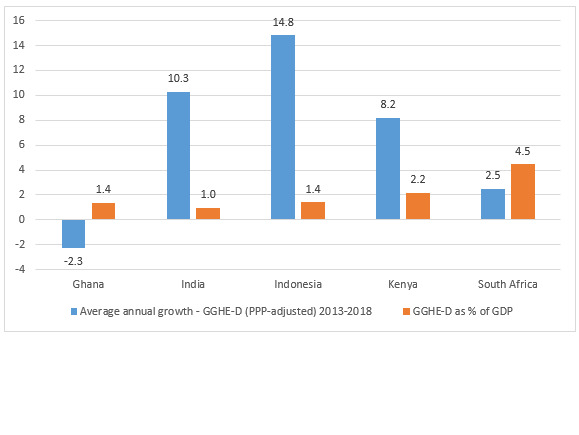
Health spending growth in five years to 2018 and domestic general government health expenditure as a share of gross domestic product (GGHE/GDP) ratio [12].

Inadequate funding was a significant barrier in Kenya, Indonesia, India and Ghana. Despite rapid economic growth, exceeding 5% per year (**Table 1** and **Figure 2**), health spending in these countries tended to be low, with domestic general government health expenditure (GGHE-D) well below notional global benchmarks, such as spending 5% of GDP [5] (GGHE-D/GDP). For example, GGHE-D/GDP was only 1% of GDP in India and 1.4% in Ghana and Indonesia, whereas GGHE-D/GGE (the health sector’s share of government expenditure) was only 3.4% in India and 6.4% in Ghana). This was associated with high out of pocket payments (eg, 62.7% India, **Table 1**) and low levels of financial protection for significant parts of the population that are not adequately covered. In these four countries, national revenue systems are incompletely developed, with a low ratio of total government revenue to GDP (<20% in three countries), likely contributing to high fiscal deficits. Despite additional revenue streams in Ghana (VAT, social security contributions), public health spending declined in the five years to 2018.

Health expenditure was nevertheless rising in some of the countries, particularly those with fast growing economies. In Indonesia, such health spending increased by 14.8% per annum (PPP adjusted) in the five years to 2018 and in India by 10.3% per annum. This rapid health spending growth was from a low base (**Figure 2** and **Table 1**). In contrast, health spending growth for Ghana (-2.3%), and South Africa (2.5%) has been negative or low.

In several of the countries low levels of health financing translated into low levels of key health inputs, such as doctors and nurse ratios per 10 000 population. For example, Ghana had only 1.3 and Kenya 1.6 doctors per 10 000 population, as compared for example to 34 per 10 000 in the Organisation for Economic Co-operation and Development (OECD) [14].

### Financial and risk pooling: fragmented pooling arrangements contribute to incomplete coverage

Several of the country case studies examined financial pooling arrangements as one of the key elements in making progress towards UHC, especially in ensuring effective coverage and achieving health sector goals. All five countries have embarked on far reaching pooling reforms, such as the Jaminan Kesehatan Nasional (JKN) Insurance scheme in Indonesia, Ayushman Bharat PM-JAY in India and the NHI scheme in Ghana. The setting up of UHC agencies, and pooling the revenues or reducing fragmentation, provide powerful tools for advancing strategic purchasing goals such as economies of scale (leading to lower costs), integrated information systems, prospective and budget-neutral provider payments and benefits packages aligned with national health priorities and cost-effective interventions. However, in this set of countries pooling reforms are not yet complete and are often associated with incomplete coverage.

Of the five countries, **Indonesia** has made the greatest progress on pooling. With the aim of providing universal health coverage to all Indonesians by 2019, landmark legislation in 2004 [15] and 2011 [16] has helped Indonesia develop one of the largest single-payer social health insurance programs - JKN. It had four main pre-existing schemes: 1) Askes – for civil servants set up at the state/province level; 2) Jamsostek – for private sector set up at the state/province level; 3) Jamkesmas – a national scheme for poor and near poor set up by central govt; and 4) Jamkesdas – local health insurance schemes for poor and disadvantaged not covered by Jamkesmas set up at the local government level (ie, 300+ district level pools). Indonesia consolidated these numerous schemes and risk pools into one national risk pool, a uniform benefit package, and a single purchaser of health services – *Badan Penyelenggara Jaminan Sosial – Kesehatan (BPJS – K)*. As of January 2019, JKN covered 215.7 million people or around 81 percent of the total population. While the premium for the poor is completely covered through a public subsidy - *Penerima Bantuan Iuran (PBI),* the remaining population pays for the premium through payroll or voluntary contributions (for informal sector non-poor), though participation is mandatory as per the law.

The Government of **India** and various state governments have introduced demand-side financing mechanisms to provide financial security for vulnerable segments of the society. These schemes started mostly from 2007 onwards, and by 2018, more than 25 such schemes were being implemented by Central and State Governments with varied benefit packages and target population. All these schemes focused only on inpatient services, and the majority only provided high end tertiary care cover.

Realising the limitation of focusing only on inpatient cover and to remove such huge fragmentation of different schemes across the country, the Government of India launched the *Ayushman Bharat* (Long Live India) programme in 2018 [17], which, in a significant departure from previous efforts, provided comprehensive coverage including primary, secondary, and tertiary care. The first pillar of Ayushman Bharat focused on providing comprehensive primary care by setting up of 150 000 Health and Wellness Centres. This is an ambitious plan as, currently, PHC centres mostly provide maternal and child health services only.

The second pillar of Ayushman Bharat is called Pradhan Mantri – Jan Arogya Yojana (PM-JAY, also known as “Modicare”) and it provides a cover of Rs. 500 000 (US$7 210) per family per year for 107.4 million families (more than 500 million persons) for most of the secondary and tertiary care conditions making it the largest fully Government-funded health insurance programme in the world. The objectives of the scheme are to reduce catastrophic out-of-pocket-expenditure, improve access to quality health care and reduce unmet needs.

One of the biggest strengths of the PM-JAY is its flexible design which considers the federal nature of the country where health is a “State subject” under the Constitution. These flexibilities include the implementation model; benefits packages and their pricing; reservation of packages for public hospitals; the IT system; and optional expansion of coverage beyond the poorest 40 percent covered by PM-JAY. Out of 36 States/Union Territories, 32 have already joined the scheme.

Since PM-JAY covers only the poorest 40% of the population, States are free to expand the coverage to additional sets of families. A few States have even universalised the coverage. Many others have expanded coverage to 50%-80% of the total population. After such expansion by the States, PM-JAY and States schemes together cover more than 700 million people out of 1.3 billion persons, and an additional 150-200 million are covered by schemes for organised sector workers. Therefore, there is still a large population of 300-350 million which is not covered by any health insurance/assurance scheme.

**Ghana’s** NHIS was introduced in 2003 (under Act 650, later amended by Act 852 in 2012) to replace the “cash-and-carry” system with a more equitable financing system with funding largely drawn from public taxes. It was to be financed by an earmarked (VAT-based) levy, part of workers’ social security contributions and premiums from informal sector adults. A single purchaser, the National Health Insurance Authority (NHIA), was empowered to run a single national health insurance fund, into which all its funds are paid, thus creating a potentially powerful strategic purchaser for the benefits package of the NHIS.

Though the NHIS showed early promise, its initial population coverage growth has since stagnated at less than 40 percent, partly linked to chronic funding shortfalls and expensive fee-for-service reimbursement mechanisms. Among the NHIS’s positive design features, the scheme promises a uniform benefit package to all members irrespective of their contribution levels and to which sector they belong (formal or informal). Residual fragmentation, contributing to inefficiencies, can however be seen in how salaries and capital costs of the public sector facilities and some vertical programs are still paid directly by the Ministry of Finance and donors.

**South Africa** has been one of the slowest of this group of countries to reform its pooling arrangements. Despite over a decade of policy proposals for introduction of NHI, its health system remains almost entirely separated into public and private financing systems, in which only 17% of the population hold private health insurance [18] cover which spends close to 50% of total expenditure on health [12]. The slowness of reform in South Africa is related to many factors including management and governance weaknesses [19].

**Kenya’s** draft health financing strategy proposes to establish funds at national and county levels for managing government and external resources and creation of a Kenya Social Health Insurance Fund (SHIF) for pooling and strategic purchasing with mandatory and voluntary insurance contributions. Government envisages the existing National Hospital Insurance Fund (NHIF), established in 1965, to be the ultimate vehicle for implementation of UHC. The NHIF is currently the major health insurance provider in Kenya covering 89% of insured (about 20% of total population), mainly those working in the formal sector. The NHIF has been made into a state corporation mandated to pool formal sector employee contributions made on a graded scale and undertake strategic purchasing of hospital services which was extended to include outpatient benefits. The free maternity care and insurance cover for the elderly also operate through NHIF, reducing fragmentation. However, contributions by informal sector are still low with high dropout rates and contribution by employers is not mandated.

### Strategic purchasing

In many of these countries strategic purchasing holds particular relevance, because of a long history of inputs-based financing and lack of accountability for results in their health sectors. Properly deployed, strategic purchasing can enable significant progress towards achieving key health sector objectives such as efficiency, equity, sustainability, accountability, and user satisfaction.

The design of **Ghana**’s NHIS, as noted, confers significant strategic purchasing potential on the scheme. Some of those advantages are evident. For example, the NHIA credentials public and private providers, from whom members receive health care, by ensuring they undergo the necessary quality assurance checks and review processes as required by the NHIS protocols. Those protocols aim to ensure that providers who are credentialed have satisfied the NHIA that they can provide safe, efficient, effective, and quality services.

But in a larger sense, the strategic purchasing potential of the NHIS has remained, for the most part, unrealised. The NHIS has been held back partly by its reliance on fee-for-service reimbursement and DRG payment mechanisms. But the NHIS also seems burdened with a benefit package that may be maladapted for the county’s stage of development. Public dissatisfaction is reflected in a widespread perception that the quality of services provided under the NHIS is not good [20].

A seven-member technical committee (for full disclosure, the author of the Ghana case study in this paper was the chair of this committee) appointed by the President of Ghana in September 2015 to review the NHIS noted the benefit package’s curative care focus which helps to disincentivize utilization of essential PHC services that are more directly related to the country’s low performance on key health indicators. It suggested refocusing the scheme primarily (but not exclusively) around achieving PHC for all Ghanaians (including essential preventive and promotive care) as the best use of the scarce NHIS resources [21].

**Indonesia,** after consolidating its numerous risk pools, defining a uniform benefit package, and setting up a single purchaser of health services, still has a mixed record in strategic purchasing and achieving the desired results. The JKN offers a generous open-ended benefits package, with a few exceptions as part of a negative list, but actuarial estimates indicate that the program is under-resourced for the generous benefits package it offers. Currently, it also has no co-payments or global caps on hospital expenditures, which is where the bulk of the expenditures ( ~ 80%) occur. Given this, the program has been facing an increasing financial deficit since its inception, which is expected to grow further, if unaddressed [22]. Also, short activation periods for new or returning members and poor contribution compliance further encourage members to only sign up when sick and stop paying once treatment has been received.

**India**’s PM-Jay reforms have created a unified but flexible national health financing system, drawing strength from enabling States to modify and own key design features affecting implementation of purchasing and other arrangements. States have taken advantage of the opportunities to vary the implementation of the reforms to suit their preferences.

PM-Jay provides approximately 1400 pre-defined and pre-costed benefit packages. However, as States can add additional benefit packages, 15 States have revised the package list. States can even revise the rates for the packages and 8 have accordingly revised the package prices.

The National Health Authority (NHA) has reserved a few packages for public hospitals based on potential for misuse. States are also free to reserve defined packages for public hospitals depending upon the strength of the public hospitals and potential for misuse. Most States have used this flexibility.

PM-JAY also provides a standard IT system to all the States. However, States are free to use their own IT system. 10 States are using their own IT systems. In these cases, States only need to provide defined data in a standard format to the NHA.

**Figure 3:**
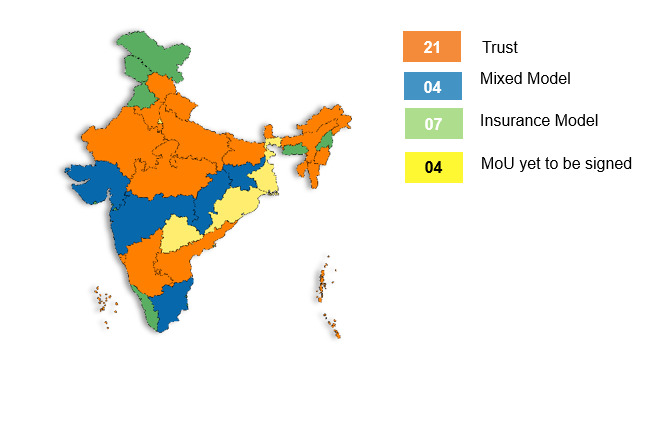
Implementation models across Indian States and UTs [17].

The scheme gives the freedom to the State in deciding the implementation model (**Figure 3**). States can use an “Insurance” model (engage an insurance company to purchase health care services), a “Trust” model (purchase services directly by the State-owned agency) or a “Mixed” model (use insurance company for one part and trust for the other part).

**Kenya** has a small-sized National Health Insurance system and limited strategic purchasing. Accredited providers from public, faith-based and private sectors are contracted by the NHIF, but salaries and allowances for public providers are paid directly to their bank accounts and not linked to performance. Public hospitals are mandated by the PFM Act to deposit their revenues from both user fees and reimbursements from NHIF into the county revenue funds. This results in indifference among public facilities to prepare reimbursement claims for insured persons, affecting critical programs like free maternity care. Private facilities tend to be more prompt as they are directly incentivized as the provider. Although public sector and faith-based organizations have the widest coverage, a major share of NHIF payments go to private and faith-based sectors due to this difference in incentive.

**South Africa** has made little progress in strategic purchasing, with public purchasers utilising almost no services from the large private sector and not using strategic purchasing within the public sector either [19]. Fee-for-service remains dominant as the main provider payment mechanism in the private sector, contributing to problems of high prices and supplier-induced demand [23].

### Governance, institutional arrangements, and decentralization

Most of these five countries have complex decentralised systems and the variability across decentralised states or provinces considerably affects UHC reforms. These include powerful states in India, provincial governments in South Africa and counties in Kenya. The political will to put the drive towards UHC at or near the top of the country’s developmental agenda is a crucial element of this governance dimension, as such a will is essential for the country to surmount the many challenges and obstacles that will arise on the UHC journey.

In **Indonesia**, decentralization has strained the limited capacity of local governments to do integrated health planning and budgeting, compounded by multiple and fragmented financing lines and poor data quality and use. While the bulk of government health expenditure occurs at the district level, the central government remains the dominant source of revenues. Complex and fragmented inter-fiscal government transfers in a decentralized system resulted in wide variations of health spending across districts [24]. Also, these transfers are not performance-oriented to influence districts to allocate resources to achieve better health system results. District health offices rarely plan the use of this funding in a holistic manner to address gaps in inputs for health services, focused on populations and diseases of greatest need.

At the central level, key institutional challenges remain, including strengthening governance and accountability mechanisms for the whole sector and allocation of functions between MoH and BPJS-K to improve strategic purchasing. While the BPJS-K is increasingly becoming a purchaser of health services, many strategic purchasing functions such as determining the benefits package, provider payment mechanism and rate setting are in practice with the MOH. Similarly, quality of care is seen as the responsibility of both MoH and BPJS-K but the mechanisms to enforce this need more clarity. Multiple information systems and lack of interoperability not only increases workload of providers but also contributes to the low use of data for better decision making and program management.

In **South Africa**, decentralisation has emerged as a potential stumbling block to the specific policy trajectory of the South African NHI reform, creating a barrier to pooling in the public sector. An even more difficult step is bridging the public and private financing divide. The NHI policy is strongly predicated on a single national NHI Fund [25], while the National Health Act of 2003 [26] makes delivery of health services a provincial health function. In terms of the Constitution [27], funding follows the legal location of the function, and thus most funding for health flows to provincial governments with limited conditions attached to it. This effectively has given both funding and control of health delivery to South Africa’s nine provinces, with widely varying quality of care, something which the national Minister of Health finds difficult to be held accountable for. The potential contradiction between the single national NHI Fund and the legal power and funds residing with the nine provinces is a major issue, which has held back progress [19]. Powers have historically rested mainly with provincial governments and decentralisation to districts (n = 52) and institutions, eg, hospitals (n = 407), has been weak. This creates difficulties for establishing an effective purchaser-provider split and strategic purchasing for services, given limited financial and contractual ability of districts and health facilities.

In **India,** powerful semi-autonomous States substantially affect the way that PM-Jay is rolling out, with national frameworks and incentive structures being taken up in different measures and ways by each state government, as pointed out above.

In **Ghana**, the NHIS Act set up the scheme, the NHIS, with an NHIA as its management and regulatory unit, under the supervision of a Board of Directors appointed by the President. The NHIS was initially designed as a decentralized system with semi-autonomous board-run District Mutual Health Insurance Schemes (DMHIS) as the basic structure where individuals enrolled to become members of the NHIS. But the autonomous DMHIS were abolished under Act 852 and the NHIS is now highly centralised with little responsibility resting with its district offices.

In **Kenya**, the responsibility to deliver essential health services is decentralized to 47 county governments. The NHIF has established offices at the county level to improve enrolment of informal sector and ensure more efficient payments. However, the representation of county governments in its Board which provides voice to decentralized health system needs attention.

## CONCLUSIONS AND LESSONS

Five case studies have been presented of countries with varying and incomplete progress towards UHC. The cases focused on examining their financing, pooling, strategic purchasing, and the governance /institutional arrangements for achieving UHC. Although nearly all of these countries have made good progress on at least one dimension of UHC, there remain key barriers to further progress. The following is a summary of the major conclusions and lessons from these case studies.

***Financing:*** In several of the cases, inadequate public funding for health remains a key issue, for example in Ghana (despite dedicated sources of taxation, suggesting that ringfenced revenue for health is by no means a panacea for improving funding levels), India and Indonesia. In some cases, low levels of funding result both from weak government systems of taxation and lower prioritisation of health in the budget. Funding limitations translate into low levels of key inputs such as doctors and nurses per 1000 population. However, with rapid economic growth, health spending in Indonesia and India is increasing rapidly from a low base.

***Strategic purchasing, pooling and service coverage improvements:*** The studies highlight the need to pay attention to both supply side and demand side factors in UHC reforms: countries like Ghana, Kenya, and India which are struggling with inadequate service delivery capacities and quality of care issues, are also having difficulty making their UHC schemes attractive to the population, even with an ostensibly extensive and generous benefits package as in Ghana’s case.

Indeed, the low UHC coverage indices of most of these countries are often a reflection of ‘generous’ benefits packages on paper but which are in practice not fully available to the beneficiaries, especially those in rural and peri-urban areas. A lesson here is that substantial supply-side investments are essential to making progress on delivering the full promise of UHC to its intended beneficiaries. Most of the countries have made slow progress in using public funds to purchase from a mix of public and private delivery platforms. *Ayushman Bharat* in India has perhaps recently been most innovative in this regard, while countries such as South Africa still have poorly aligned public and private sectors with almost no strategic purchasing (despite the intended NHI policy reform).

Potentially massive pooling reforms are taking place in some of these countries such as JKN in Indonesia and *Ayushman Bharat* in India. These are expanding rapidly but, in most cases, there are still incomplete coverage levels especially for the informal sector. In South Africa, pooling reforms such as National Health Insurance (NHI) have been slow to evolve despite numerous policy papers.

Well-designed and managed information systems can greatly facilitate efficient administration of UHC schemes and thus aid the achievement of UHC goals. India provides an example of a good information system design with a standard IT system for all states, while Ghana and South Africa still have ways to go.

***Governance and institutional arrangements:*** Four countries in this group have complex decentralised systems, such as counties in Kenya, provinces in Indonesia and South Africa, and states in India, which can be a source of strength if these encourage local ownership, commitment, and participation, but can also make reform slower to achieve given variability across multiple decentralised units.

***Overall:*** Adequate financing is a major constraint for making progress towards UHC in most of these countries, which is sometimes aggravated by sub-optimal pooling and strategic purchasing arrangements and institutions. But a hopeful sign is that, while complex federal systems may complicate the path forward for most of these countries, there is clear evidence of strong political will and commitment to UHC from the governments of these countries.
